# Tejocote Root's Role in Symptomatic Mobitz Type 1 Heart Block: A Compelling Case Report

**DOI:** 10.7759/cureus.45228

**Published:** 2023-09-14

**Authors:** Heabah Assi, Carolina Najera, Omar Aboudawoud, Sahithi Nadella, Jared J Bies, Mariam Hassan, Chanwit Roongsritong

**Affiliations:** 1 Internal Medicine, Texas Tech University Health Sciences Center El Paso Paul L. Foster School of Medicine, El Paso, USA; 2 Cardiology, Texas Tech University Health Sciences Center El Paso Paul L. Foster School of Medicine, El Paso, USA

**Keywords:** digoxin, cardiovascular toxicity, herbal medicine use, hawthorn, mobitz type 1 av block, tejocote root

## Abstract

The clinical presentation and diagnosis of Tejocote root toxicity causing Mobitz Type 1 remains a scarcely clinical phenomenon, often resulting in delayed diagnosis and treatment. This case report highlights a 30-year-old female presenting with a constellation of symptoms, including fatigue, dizziness, chest pressure, myalgias, nausea, vomiting, and peripheral tingling. Significantly, the patient had been using Tejocote root as an over-the-counter laxative acquired from Mexico. Laboratory findings revealed detectable Digoxin levels in her bloodstream, while an electrocardiogram (EKG) indicated sinus bradycardia with Mobitz Type 1 heart block. The patient was treated with a single dose of atropine 0.5 mg IV push. A repeat EKG before discharge showed resolution of the Mobitz type 1. This case underscores the potential cardiovascular repercussions of Tejocote root consumption and emphasizes the importance of heightened clinical awareness, especially in regions where such herbal supplement usage is prevalent.

## Introduction

Mobitz Type 1 second-degree atrioventricular (AV) block, also known as Wenckebach AV block, is an abnormality in the transmission of the electrical signals between the atria and the ventricles [1]. This abnormality can be identified on the ECG by the progressive prolongation of the PR interval until the beat drops. When an atrial impulse is entirely blocked, a P wave will appear without a subsequent ventricular depolarization (QRS) complex [1]. After the dropped beat, the cycle repeats itself. This pattern gives rise to the term Wenckebach phenomenon [2]. There are multiple causes of reversible Mobitz type 1, including reversible ischemia, myocarditis, increased vagal tone, post-cardiac surgery, and medications that slow AV node conduction (beta-blockers, non-dihydropyridine calcium channel blocks, adenosine, digitalis, and amiodarone) [1,3]. One of the lesser-known causes of Mobitz type 1 is Tejocote (Crataegus mexicana) root ingestion. We report a case of Tejocote root ingestion causing symptomatic Mobitz type 1. 

## Case presentation

A 30-year-old female with a past medical history of an unspecified childhood murmur presented to the emergency department with complaints of lifestyle-limiting fatigue and dizziness for two weeks. These symptoms subsequently became associated with chest pressure on exertion, lightheadedness, and myalgias over the course of three days. This patient denied being on any medications for chronic medical conditions but endorsed taking several over-the-counter supplements, including vitamin C, magnesium, and tejocote root extract, which she had been utilizing as a laxative. A review of systems on admission further revealed nausea, vomiting, and a tingling sensation in her hands and feet that developed alongside the chest pressure. She denied any history of smoking or recreational or illicit drug use and endorsed only occasional alcohol intake (once or twice per year).

**Figure 1:**
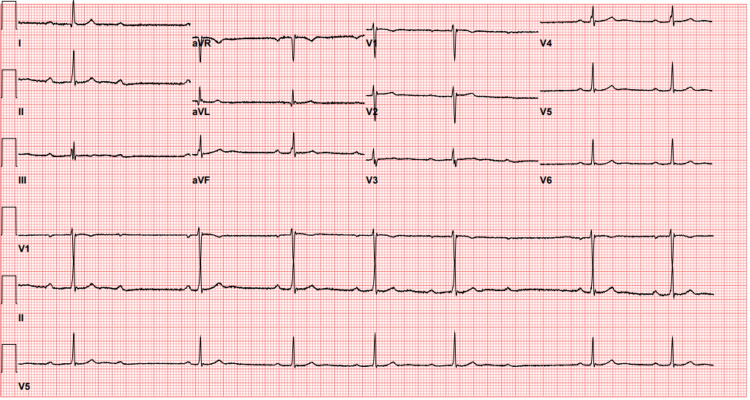
Initial EKG showed sinus bradycardia with a 2nd-degree AV block. Ventricular Rate = 42 beats per minute; QRS interval = 84 ms; QT/QTc = 388/323 ms

On admission, there was notable bradycardia, with the patient’s peripheral pulse rate ranging between 40 and 53 bpm. Blood pressure on admission was 114/74 mmHg, with subsequent blood pressure measurements demonstrating decreased diastolic pressures between 52 and 56. The patient's oxygenation status remained stable and >94% on room air throughout admission. The patient was given a single dose of atropine (0.5 mg) via IV push. On the initial physical exam, irregular bradycardia was auscultated on the chest examination. EKG on admission demonstrated sinus bradycardia with Mobitz type 1 heart block (Figure 1). 

A follow-up EKG stress test was performed, which showed adequate chronotropic response and ventricular bigeminy once heart rate reached 120 with premature ventricular beats and right ventricular outflow tract morphology. This was deemed a benign finding, given the lack of symptoms while performing a daily exercise routine prior to hospitalization. Further work-up included a TSH of 1.17 (0.465-4.68) and a troponin level of <0.012 (<0.012). A basic metabolic panel was obtained on admission (Table 1).

**Table 1:** Basic metabolic panel on admission BUN: blood urea nitrogen, A/G ratio: albumin/globulin

	Value	Normal Range
Sodium	138 mmol/L	135 to 145
Potassium	4.4 mmol/L	3.5 to 5.1
Chloride	107 mmol/L	98 to 107
Bicarbonate	26 mmol/L	22 to 30
Anion Gap	5 mmol/L	5 to 19
Glucose	91 mg/dL	74 to 106
BUN	9 mg/dL	7 to 17
Creatinine	0.7 mg/dL	0.52 to 1.04
Estimated GFR	119 ml/min/1.73 m^2^	
Calcium	8.6 mg/dL	8.4 to 10.2
Magnesium	2.0 mg/dL	1.6 0 to 2.3
Albumin	3.9 G/DL	3.5 to 5.0
Protein	6.2 G/DL	6.3 to 8.2
Total Bilirubin	0.4 mg/dL	0.2 to 1.3
Calc Osmol	284 mosm/kg	281 to 303
A/G Ratio	1.7	1.4 to 1.6

Due to the patient admitting she had obtained tejocote root extract in Mexico, further research was done on its herbal use, which prompted an evaluation of digoxin levels. Although low, a falsely elevated level of digoxin of 0.5 ng/mL was present in the laboratory workup. A formal trans-thoracic echocardiogram demonstrated a normal left ventricular ejection fraction with a grossly normal right ventricle; however, the study was overall poor in quality due to breast implant shadow.

**Figure 2:**
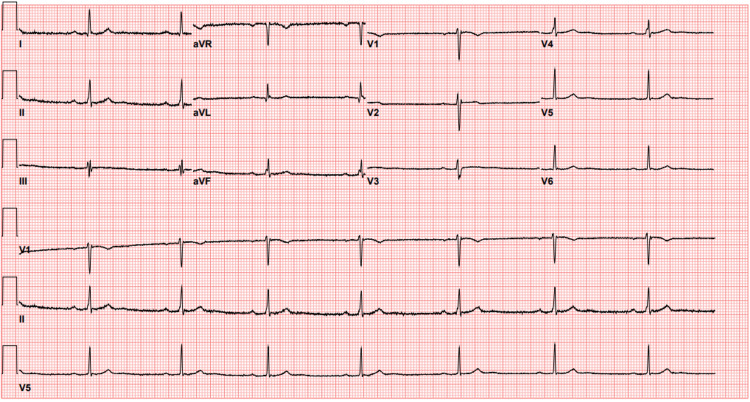
Repeat EKG showing resolution of second degree AV block, but now showing sinus bradycardia with first degree AV block. PR Interval = 222, Ventricular Rate = 45 beats per minute, QRS Interval = 82 ms, QT/QTc = 394/340

The patient was subsequently discharged in stable condition with recommendations to follow up with cardiology as an outpatient. She was asymptomatic by the time of discharge, with resolution of Mobitz type 1 on the EKG (Figure 2). 

## Discussion

A common species of hawthorn found in Mexico is Crataegus mexicana, with the fruit of this tree known as tejocote used to make various sweets such as candy and marmalades [4]. In recent years, preparations using the root have become more utilized and marketed as weight loss supplements, with the mechanism being that they lead to decreased appetite and early satiety due to the elevated pectin content [4]. However, hawthorn has been mentioned as a remedy in medicinal texts dating back to the 1st century A.D. [5]. In the 1890s, cases depicting patients suffering from various forms of heart disease and treated with hawthorn showed promising results, but it wasn’t until the 1940s that it was introduced into German pharmacopeia [5]. Hawthorn extract is known to have multiple cardiovascular benefits due to its positive inotropic effects [6]. It has been found that exercise tolerance was significantly increased, shortness of breath and fatigue improved, and the physiologic outcome of maximal workload was more beneficial for patients taking hawthorn extract who have chronic heart failure [7]. Tejocote root is a species of hawthorn that contains digitalis-like compounds that may interfere with serum digoxin measurement and cause falsely elevated digoxin levels [6,8]. Tejocote root and digoxin physiological effects may be similar, as one study showed they bind to the same site [8]. Tejocote root exerts its potent effects via multiple mechanisms, including the inhibition of sodium-potassium ATPase and enhancing endothelial relaxation through nitrous-oxide-mediated mechanisms [6]. It can prolong the action potential and refractory period, demonstrating its antiarrhythmic properties [6].

While cardiovascular benefits have been extensively studied, adverse effects remain less understood [9]. The majority of side effects reported are mild, including headaches, gastrointestinal symptoms, and dizziness or vertigo [9,10]. There are rare reported incidences of tejocote root causing more serious adverse effects, such as dysrhythmias and respiratory depression [6]. Similar to digoxin, tejocote root toxicity may slow AV conduction, potentially leading to AV block [6]. Management of mild to moderate digoxin toxicity lacks established evidence-based guidelines, resulting in a range of treatment approaches [11]. Severe toxicity may necessitate the use of digoxin-specific antibody fragments, indicated in cases of life-threatening or hemodynamically unstable dysrhythmia, cardiac arrest, or when potassium levels exceed 5.0 mmol/L [11]. Additional options include activated charcoal for patients presenting within two hours of ingestion and atropine for bradyarrhythmia [11]. In our patient it presented with symptomatic Mobitz type 1 and notable bradycardia. 

A second-degree atrioventricular block can be divided into types 1 and 2. Mobitz type 1, known as Wenckenbach, is due to a delay in conduction between the atria and ventricles, causing an atrioventricular block and prolonged PR interval on the electrocardiogram [1]. It is characterized by P waves with a constant rate, usually <100 beats per minute, accompanied by a periodically dropped P wave, with the P waves before or following the dropped P wave with inconsistent PR intervals [12]. Generally, patients with second-degree AV block are asymptomatic or present with lightheadedness and syncope [1]. In symptomatic patients with Mobitz type 1, ECG monitoring can be a reasonable initial step in diagnosis for determining the correlation of symptoms with rhythm abnormalities [12]. Symptomatic patients should be admitted for monitoring and can be managed with atropine or transvenous pacing [1].

There have been previously reported cases of tejocote root toxicity presenting with cardiotoxicity and arrhythmias, as well as other rare adverse effects; however, data is limited as few cases have been reported [4,6,9,13,14] (Table 2).

**Table 2:** A list of published tejocote toxicity case reports

Case Report	Age	Gender	Country	Presentation	Treatment
Crataegus mexicana (Tejocote) Exposure Associated with Cardiotoxicity and a Falsely Elevated Digoxin Leve	16	Female	United States	Drowsiness, vomiting, diarrhea, and an ECG demonstrated a heart rate of 38 and Mobitz type 1 second-degree heart block.	Two doses of Digoxin Immune Fab
The Forbidden Fruit: A Case of Tejocote (Crataegus mexicana) Supplement Toxicity	55	Female	United States	Weakness, nausea, abdominal pain, and ECG showed bradycardia at a rate of 48 beats/min and a prolonging PR interval followed by a non-conducted QRS complex, indicative of a second-degree atrioventricular block, Mobitz type 1.	Monitored on telemetry for four days, during which time her symptoms improved.
Fatal arrhythmia following ingestion of hawthorn root (Crataegus pubescens) extract: a case report	20	Female	Mexico	Abdominal pain and nausea developed into hypotension and bradycardia, with the ECG showing sinus arrest with a slow nodal rhythm and secondary changes of the ST segment and T wave that progressed to depressed consciousness and cardiac arrest.	N/A
An Atypical Etiology of Acute Pericarditis	23	Female	United States	Chest pain radiating to her back was exacerbated by lying down, fatigue, and loose stools, and based on her symptoms and an ECG finding of T-wave inversions, the patient was diagnosed with acute pericarditis.	She was started on intravenous Toradol (15 milligrams every eight hours) and colchicine (0.6 milligrams daily) and discharged on a three-month course of colchicine.
Immune thrombocytopenic purpura caused by over-the-counter weight supplement Root of Tejocote (Crateggus species)	51	Female	United States	Generalized malaise for one month with a hemogram revealing new thrombocytopenia.	Treated with a prednisone taper that was gradually reduced by 10mg weekly and follow-up outpatient.

## Conclusions

This case highlights the importance of considering reversible causes of Mobitz type 1 second-degree atrioventricular (AV) block. The patient, in this case, presented with symptoms of fatigue, dizziness, chest pressure on exertion, and myalgias, with the onset of symptoms consistent with the use of Tejocote root extract as a laxative. The patient's EKG on admission demonstrated sinus bradycardia with Mobitz type 1 heart block, and subsequent work-up revealed a detectable level of Digoxin, suggesting a possible contributing factor to the AV block. It is important for clinicians to remain vigilant of the potential cardiac effects of herbal and supplement use, especially in regions where such practices are widespread, as they can significantly impact patient care. Despite the limited literature on Tejocote root’s clinical effects, further studies are needed to elucidate the mechanisms underlying Tejocote root ingestion as a reversible cause of Mobitz type 1 and to develop strategies for prevention and treatment.
